# Nephrotic Syndrome Induced by Lenvatinib Treatment for Hepatocellular Carcinoma

**DOI:** 10.1155/2022/5101856

**Published:** 2022-09-05

**Authors:** Thaninee Prasoppokakorn, Kessarin Thanapirom, Sombat Treeprasertsuk

**Affiliations:** ^1^Division of Gastroenterology, Department of Medicine, Faculty of Medicine, Chulalongkorn University, Bangkok, Thailand; ^2^Center of Excellence in Liver Diseases, King Chulalongkorn Memorial Hospital, Thai Red Cross Society, Bangkok, Thailand; ^3^Liver Fibrosis and Cirrhosis Research Unit, Chulalongkorn University, Bangkok, Thailand

## Abstract

Lenvatinib, an oral small-molecule multiple tyrosine kinase inhibitor (TKI), has been approved for first-line therapy for unresectable hepatocellular carcinoma (HCC). Proteinuria is one of the most common adverse events associated with lenvatinib treatment. We reported a 67-year-old Thai female was diagnosed with NASH cirrhosis and HCC BCLC B with TACE refractoriness. She received 8 mg of lenvatinib for 2 weeks and began to experience worsening hypertension, bilateral pleural effusion, pedal edema, hypoalbuminemia, hypercholesterolemia, and proteinuria. After exclusion of all possible causes, lenvatinib-induced nephrotic syndrome (NS) was diagnosed. One week after discontinuing the drug, her symptoms gradually improved. To date, there have been only a handful of reported cases of lenvatinib-induced nephrotoxicity. We report herein the case of lenvatinib-induced NS in a cirrhotic patient with HCC with resolution of symptoms in a short period after drug discontinuation. In addition, we reviewed all reported cases of lenvatinib-induced nephrotoxicity.

## 1. Introduction

Lenvatinib is an oral small-molecule multiple tyrosine kinase inhibitor (TKI) that inhibits vascular endothelial growth factor receptor 1–3 (VEGFR1–3), fibroblast growth factor receptor (FGFR1-4), platelet-derived growth factor receptor (PDGFR), stem cell factor receptor (KIT), and rearranged during transfection (RET) [1, 2]. It has been approved for first-line therapy for unresectable hepatocellular carcinoma (HCC) [3–5]. A recent randomized controlled study showed that it was noninferior to sorafenib in terms of overall survival (OS), but was associated with: longer progression-free survival, longer time to progression, and higher objective response rate in comparison to sorafenib based on results of REFLECT trial [6]. The most common adverse events of lenvatinib are hypertension, diarrhea, decreased weight and appetite, and proteinuria. We reported herein a cirrhotic patient with unresectable HCC who received lenvatinib and developed nephrotic syndrome due to lenvatinib treatment.

## 2. Case Report

**Figure 1:**
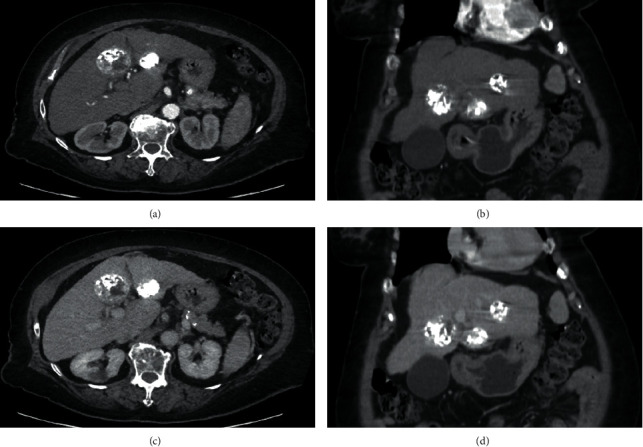
Abdominal computed tomography revealing a cirrhotic liver with 3 partial lipiodol-staining masses at left hepatic lobe with remaining viable part, size 3.7 × 2.9 cm at segment III (viable part at anteroinferior aspect), 4.5 × 4.4 cm at segment IVb (viable part at right posterolateral aspect), and 2.7 × 2.5 cm at segment II (small viable part at posterior aspect) in axial and coronal view of arterial phase (a-b) and axial and coronal view of portovenous phase (c-d), respectively.

A 67-year-old Thai female was hospitalized due to worsening dyspnea and orthopnea for 1 week. She was previously diagnosed with nonalcoholic steatohepatitis (NASH)-related Child–Pugh A cirrhosis (6 points) and Barcelona Clinic Liver Cancer (BCLC) stage B HCC 6 months previously. Abdominal computed tomography (CT) revealed a cirrhotic liver with many arterial enhancing lesions in both hepatic lobes with rapid wash-out in portal and delayed phases. The largest tumor size of 4.0 × 4.2 × 4.5 cm in segments V/VII indicated multifocal HCC with the absence of portal vein invasion and thrombosis. She received locoregional therapy for HCC by transarterial chemoembolization (TACE) 2 times but failed to experience significant response. CT at 1-month follow-up after TACE demonstrated a viable tumor 3.1 × 2.9 cm at hepatic segment V (Figure 1). After discussion with patient for further treatments, she was switched to systemic therapy using a molecular targeted agent. An 8 mg daily dose of lenvatinib was prescribed 2 weeks before this hospital admission. Her medical history included well-controlled hypertension, dyslipidemia, type 2 diabetes mellitus, and Graves' disease treated with radioactive iodine.

Physical examination revealed body temperature of 36.2°C, blood pressure (BP) of 190/120 mm·Hg (baseline BP 110/60), pulse rate of 110/min, respiratory rate of 24/min, puffy eye lids, decreased breath sounds and dullness on percussion at lower half of the right lung, and bilateral pedal edema.

**Figure 2:**
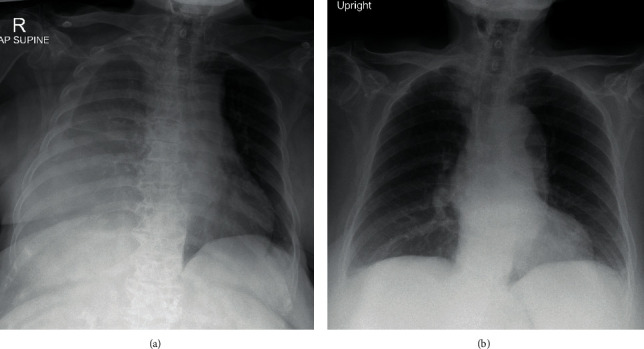
Chest X-rays revealing moderate amount of right pleural effusion with pressure effect caused trachea shift to the left (a) and complete resolution of right pleural effusion after discontinuing lenvatinib for 1 week (b).

Chest X-ray (CXR) showed moderate right pleural effusion (Figure 2(a)). Pleural fluid analysis exhibited white blood cell (WBC) of 111 cells/*µ*L (neutrophil 28%; lymphocyte 72%), protein of 1.5 g/dL, lactate dehydrogenase of 78 U/L, and no organism on Gram stain and culture indicating transudative profile. Serum biochemical tests showed total bilirubin of 1.2 mg/dL, direct bilirubin of 0.6 mg/dL, SGOT of 50 U/L, SGPT of 34 U/L, alkaline phosphatase of 99 U/L, BUN of 29 mg/dL, creatinine of 0.9 mg/dL, eGFR of 66 mL/min/1.73 m^2^, albumin of 2.0 g/dL, protein of 6.7 g/dL, LDH of 238 U/L, and cholesterol of 260 mg/dL. Urinalysis showed red blood cell and WBC of 0-1 cell/high power field (HPF) and 3^+^ albumin. The 24-hour urine albumin was 7.01 g/day. The thyroid function test was normal. The diagnosis of nephrotic syndrome (NS) was definitive. After exclusion of other possible causes of NS including HIV, HBV, HCV, and autoimmune diseases, the diagnosis of lenvatinib-induced NS with worsening hypertension was made. Lenvatinib was immediately discontinued due to this serious adverse event.

One week after discontinuation of lenvatinib and supportive therapy, the patient symptoms gradually improved with a reduction of right pleural effusion (Figure 2(b)) and lowering of the urine protein creatinine ratio (UPCR) from 9.7 to 5.6 g/day after 2 weeks. After discussion with the patient and her family, the decision was made to give supportive care for her HCC for a while. Six months after stopping lenvatinib, UPCR declined to subnephrotic range proteinuria of 0.95 g/day as well as 24-hour urine albumin was only 0.54 g/day. Due to the patient having good performance status as well as preserved liver function, the second line systemic therapy was prescribed with 180 mg of nivolumab. The patient had well-tolerated to treatment with 7 months courses of stable disease by follow-up imaging. Besides, proteinuria was closed follow-up every outpatient visit and demonstrated no evidence of recurrence.

## 3. Discussion

There have been very few reported cases of lenvatinib-induced nephrotoxicity published in medical journals. We reported herein a case of lenvatinib-induced NS in a cirrhotic patient with HCC, which developed within 2 weeks after beginning a course of 8 mg lenvatinib. Resolution of symptoms began soon after discontinuation of medication.

Among molecular targeted agents, lenvatinib is a TKI that inhibits many growth factors and is prescribed for the treatment of many types of cancers including thyroid cancer and HCC. The most common adverse events associated with lenvatinib include hypertension (42%), diarrhea (39%), decreased appetite (34%), decreased weight (31%), and proteinuria (25%) [6].

Preclinical studies in human HCC models have reported that lenvatinib has potent antiangiogenic activity, mainly through the pathways of VEGFR and FGFR inhibition [7]. The VEGF pathway regulates angiogenesis which is an essential event in tumor growth and metastasis which provides oxygen and nutrients to tumor cells. Targeting the VEGFR-mediated pathway can lead to starvation, hypoxia, and tumor cell death [2].

Proteinuria associated with angiogenesis inhibition is the most common renal side effect of anti-VEGF drugs with a prevalence ranging from 21 to 63% and frequently occurring with hypertension. Due to VEGFR binding in glomerular and peritubular endothelium, as well as in mesangial cells in normal conditions, this binding process plays a role in the maintenance of the glomerular basement membrane structure and proper glomerular function. The drugs block VEGFR and may induce renal abnormalities, especially in renovascular function that includes hypertension and proteinuria. Moreover, there have been reported patients developing renal thrombotic microangiopathy (TMA) cause by VEGF trap and multitargeted TKI [9]. The presentative symptoms can be mimicked with nephrotic syndrome with renal dysfunction [9]. The drugs have the potential to cause nephrotic syndrome. Potential mechanisms of lenvatinib-induced proteinuria include postexercise proteinuria-like syndrome, perturbation of podocyte-endothelial VEGF axis signaling, podocyte protein junction downregulation, subacute glomerular thrombotic microangiopathy, and additional pathogenic factors such as hypertensive injury [10]. The exact mechanism and factors associated with the occurrence and severity of proteinuria are unknown [11, 12]. According to the reported studies in patients with thyroid cancer, lenvatinib-induced proteinuria can be observed at any time after drug initiation. The mean duration of the development of renal adverse effects after lenvatinib initiation was 43 (range 5–799) days [13–16]. Management should be dose reduced to minimize the risk of renal adverse events or complete drug discontinuation. Data showed that patients can recover within 9 weeks after stopping lenvatinib [14].

The maximum recommended dose of lenvatinib for HCC treatment is 12 mg per day, whereas 24 mg per day can be prescribed for radioiodine-refractory thyroid cancer [13]. The half-dose reduction of lenvatinib for HCC treatment may be due to the fact that lenvatinib is metabolized in the liver [17].

Management of adverse events includes supportive care, dose modification, and discontinuation of lenvatinib in cases with grade 4 adverse events. Clinical practice management of adverse events from lenvatinib-induced proteinuria according to the Common Terminology Criteria for Adverse Events (CTCAE) are listed in Table 1 [12, 17–20]. Regarding proteinuria, lenvatinib can induce renal failure defined as a decline of eGFR in the long-term analysis. They might be associated with glomerular damage, with secondary proteinuria and tubular injury, especially with long-term lenvatinib used duration over 2 years [21, 22].

Our patient rapidly developed NS just 2 weeks after lenvatinib treatment which was faster than other reported cases. Multiple patient comorbidities, including long-standing hypertension, diabetes, dyslipidemia, old age, and decompensated cirrhosis, likely predisposed the patient for the rapid development of NS. The limitation of our reported patient was an absence of definite diagnosis by renal biopsy. However, despite the lack of histopathologic findings from renal parenchyma, the diagnosis of lenvatinib-induced NS was suspected due to the improvement of patient symptoms and reduction of proteinuria following discontinuation of lenvatinib within 1 week [19].

In addition, we retrospectively reported a literature review. A total of eight patients with lenvatinib-induced renal adverse effects have currently been reported in the literature (Table 2) [16, 22–25]. Cases consisted of 3 males and 5 females with the mean age of 59.8 ± 13.3 (range: 36–79) years. There were 4 Asians and 4 Caucasians. Of the eight patients, thyroid carcinoma was the most common cancer (*n* = 6, 75.0%), followed by HCC and carcinoma of the minor salivary gland (*n* = 1, 12.5%). The dosage of lenvatinib ranged from 8 to 24 mg, and the duration of treatment ranged from 0.5 to 48 months. Compared to prior reported cases, our patient received the lenvatinib with the lowest dose (8 mg) and shortest duration (2 weeks). Regarding the clinical manifestations of nephrotoxicity, worsening hypertension was the most common event (*n* = 7, 87.5%), followed by edema (*n* = 5, 62.5%), renal function decline (*n* = 2, 25.0%), and pleural effusion (*n* = 1, 12.5% in our case). The 24-hour urine protein ranged from <1 to 11.8 g/day. With respect to renal pathology, focal segmental glomerulosclerosis and thrombotic microangiopathy were noted in 3 and 1 patients, respectively, while 4 patients did not complete a renal biopsy. The management in all patients (*n* = 8, 100%) was lenvatinib discontinuation. Three patients had been failed for prior dose reduction; one patient was treated with 40 mg of prednisolone which was tapered off within 6 months, and another one was treated by sorafenib replacement. The treatment outcomes for lenvatinib-induced renal toxicity were excellent: complete resolution (*n* = 2, 25.0%) and partial resolution (*n* = 6, 75.0%). The recovery duration after the discontinued use of lenvatinib ranged from 1 week to 15 months. Our case study patient reported the shortest symptoms recovery time of 1 week.

## 4. Conclusion

To date, there have been only a handful of reported cases of lenvatinib-induced nephrotoxicity. We report herein the case of lenvatinib-induced NS in a cirrhotic patient with HCC with resolution of symptoms in a short period after drug discontinuation.

## Figures and Tables

**Table 1:** Common adverse events and recommended management for lenvatinib treatment.

Adverse events	CTCAE grade	CTCAE description	Management
Proteinuria	1	0.15–1.0 g/24 h	None
	2	>1.0–3.5 g/24 h	(i) Hold lenvatinib until proteinuria resolves to ≤2.0 g/24 h (ii) Consider consult nephrology
	3	>3.5 g/24 h	(i) Hold lenvatinib until proteinuria resolves to ≤2.0 g/24 h (ii) Referral to nephrology
	4	Nephrotic syndrome	(i) Discontinue lenvatinib permanently (ii) Referral to nephrology

CTCAE, Common Terminology Criteria for Adverse Events; *g*, gram; *h*, hours.

**Table 2:** A summary of all 8 patients with lenvatinib-induced renal toxicity published in English literature from 2019 to 2020.

Patient	Gender/age (year)	Race	Cancer	Lenvatinib dose (per day)/duration (month)	Clinical presentations	Renal function: creatinine (mg/dL)/eGFR (mL/min/1.73 m^2^)	Urinalysis/24-hour protein urine (g)	Renal histological finding	Treatment	Outcome
1, 2018 [23]	F/36	Caucasian	Medullary thyroid carcinoma	24 mg/19	Worsening HT, ankle edema	Normal	NA/3.1 g	FSGS	Discontinued lenvatinib	No proteinuria in 1 year
2, 2018 [23]	F/70	Japan	Papillary thyroid carcinoma	24 mg/26	Worsening HT, legs edema	1.12/37.4	WBC <1, RBC 5–9 cells/HPF/3.5 g	TMA	Decreased dose to 8 mg of lenvatinib and discontinuation	Partial recovery in 2 months
3, 2018 [16]	F/79	Japan	Papillary thyroid carcinoma	10 mg/3	Worsening HT, generalized edema	1.17/34	RBC 20–29 cells/HPF/11.8 g	FSGS	Discontinued lenvatinib	Complete recovery in 15 months
4, 2018 [22]	M/44	Caucasian	Papillary thyroid carcinoma	24 mg/6 20 mg/4 14 mg/26 10 mg/12	Worsening HT	1.90/NA	NA/3.5 g	FSGS, tubulointerstitial vascular necrosis, TMA-like pattern	Decreased dose to 10 mg of lenvatinib and discontinued	Partial recovery in 1 month
5, 2018 [22]	F/59	Caucasian	Adenoid cystic carcinoma of the minor salivary gland	24 mg/15	Acute renal failure (tubulointerstitial nephropathy by clinics)	2.81/NA	No sediment/< 1 g	Not performed	Discontinued lenvatinib. Prednisolone 40 mg taper in 6 months	Renal function improved in 10 days
6, 2018 [24]	M/67	Caucasian	Papillary thyroid carcinoma	24 mg/9	Worsening HT and renal function	2.46/26.0	NA/NA	Not performed	Discontinued lenvatinib	Partial recovery in 8 months
7, 2020 [25]	M/56	Taiwan	Papillary thyroid carcinoma	20 mg/1	Worsening HT, legs edema	0.56/115	Trace hematuria/9.9 g	Not performed	Decreased dose to 10 mg of lenvatinib and discontinued with sorafenib replacement	Partial recovery in 5 months
8 (our case), 2020	F/67	Thai	Hepatocellular carcinoma	8/0.5	Worsening HT, bilateral pleural effusion, legs edema	0.9/66	WBC 0–1, RBC 0–1 cells/HPF/7.01 g	Not performed	Discontinued lenvatinib	Partial recovery in 1 week

FSGS, focal segmental glomerulosclerosis; TMA, thrombotic microangiopathy; HT, hypertension; AEs, adverse events; WBC, white blood cells; RBC, red blood cells; F, female; *M*, male; NA, not applicable.

## Data Availability

The data used to support the findings of this study are included within the article.

## Abbreviations

BCLC: Barcelona Clinic Liver Cancer
BP: Blood pressure
CT: Computed tomography
CTCAE: Common Terminology Criteria for Adverse Events
CXR: Chest X-ray
eGFR: Estimated glomerular filtration rate
FGFR: Fibroblast growth factor receptor
HBV: Hepatitis B virus
HCC: Hepatocellular carcinoma
HCV: Hepatitis C virus
HIV: Human immunodeficiency virus
HPF: High power field
KIT: Stem cell factor receptor
LDH: Lactate dehydrogenase
NASH: Nonalcoholic steatohepatitis
NS: Nephrotic syndrome
OS: Overall survival
PDGFR: Platelet-derived growth factor receptor
RET: Rearranged during transfection
SGOT: Serum glutamic-oxaloacetic transaminase
SGPT: Serum glutamic pyruvic transaminase
TACE: Transarterial chemoembolization
TKI: Tyrosine kinase inhibitor
TMA: Thrombotic microangiopathy
UPCR: Urine protein creatinine ratio
VEGFR: Vascular endothelial growth factor receptor
WBC: White blood cell.
